# Solubility Enhancement of a Poorly Water-Soluble Drug Using Hydrotropy and Mixed Hydrotropy-Based Solid Dispersion Techniques

**DOI:** 10.1155/2022/7161660

**Published:** 2022-11-28

**Authors:** Hezha Abdullah Ali, Hunar Kamal Omer

**Affiliations:** ^1^Department of Pharmaceutics, College of Pharmacy, University of Duhok, Dahuk, Iraq; ^2^Department of Pharmaceutics, College of Pharmacy, Hawler Medical University, Erbil, Iraq

## Abstract

**Purpose:**

The biopharmaceutics classification system places rosuvastatin calcium in class II has a low and fluctuating oral bioavailability. The research focus is to maximize rosuvastatin calcium solubility in water and dissolution rate by employing and combining various hydrotropic agents to make a solid dispersion using solvent evaporation techniques. *Methodology*. The experimental study was conducted at Duhok University, College of Pharmacy. Initially, assess rosuvastatin's solubility in hydrotropic agents including urea, mannitol, citric acid, sodium benzoate, and sodium salicylate at concentrations of 10, 20, 30, and 40% w/v. Then, various ratios of 2 and 3 hydrotropic agents were employed to reduce the concentration of each hydrotropic agent. By using a solvent evaporation procedure, solid dispersions were made. The solid dispersion powders underwent evaluation for their percentage drug content, percentage yield, solubility, dissolution test, XRD, DSC, SEM, and FTIR. For statistical analysis, GraphPad InStat Demo software was used to conduct a two-way analysis of variance (ANOVA).

**Results:**

In comparison to the pure drug, the solubility of hydrotropic solid dispersions and physical mixtures of rosuvastatin with a combination of hydrotropic agents (sodium salicylate, sodium benzoate, and urea) in the ratio of 13.33 for each increased in all formulations significantly, and all manufactured formulations' drug release ranged from 98.83 to 104.78%, indicating a noticeably higher dissolution rate.

**Conclusion:**

The concept of mixed hydrotropic solid dispersion was shown to be an original, risk-free, and cost-effective method for enhancing the bioavailability of drugs that have a low degree of solubility in water.

## 1. Introduction

In pharmaceutical formulations, poorly soluble medicines are a concern. Because many novel medicines found by combinatorial chemistry and high-throughput screening are poorly soluble, making them poor prospects for new medications, improving their solubility characteristics is a key hurdle that must be addressed. Because poorly soluble medicines have limited absorption and bioavailability, it is critical to enhance their solubility and/or dissolution rate. There have been several ways described for improving the solubility of poorly soluble medicines [1]. Poorly water-soluble medications have been made more soluble using a variety of techniques, such as the following: particle size reduction, nanonization, hydrotropy, pH adjustment, sonocrystallization, supercritical fluid process, solid dispersion, polymorphic alteration, inclusion complexation, surfactants, and liquisolid methods are just a few examples [2].

Surfactants: the traditional way to cause a difficult substance to dissolve is to lower the interfacial tension between the surface of the solute and the surface of the solvent. This allows for better wetting and interaction between the solute and the solvent. Using amphiphilic surfactants improves the solubility of drugs by lowering the surface tension between the drug and the solvent, improving wetting properties, and promoting micellar solubilization. Adjusting the pH is the easiest and most common way to make ionizable compounds more water-soluble. The ionization of a compound depends on the pH of the medium and the drug's pKa, according to the pH-partition hypothesis and the Henderson–Hasselbalch equation. Changes in the ionic environment can also cause salt to form right where it is. But this kind of salt cannot be formed from unionized compounds. The salts that are made can also change into acid or base forms in the digestive tract. Salt formation: for several decades, a way to make poorly soluble drug candidates (weak acids and bases) more soluble has been to add salt to them. Whether a drug will make good salts depends on how well it dissolves in water and how acidic or basic it is. The pH-solubility relationships also show what counterions are needed to make salts, how easily salts can break apart into their free acid or base forms, how they dissolve in different GI pH conditions, and if a common ion can affect the solubility and dissolution rate of salts. This method has a lot of potential to speed up the rate of dissolution, but it also has some problems, like the fact that it is hard to approve salts and does not work for neutral molecules. Polymeric change: polymorphs are different crystalline forms of a drug that may have different effects. Polymorphs can have different physicochemical properties, such as physical and chemical stability, shelf life, melting point, vapor pressure, intrinsic solubility, dissolution rate, morphology, density, biological activities, and bioavailability. Crystalline polymorphs can be stable, unstable, or metastable. Metastable forms have more energy and a larger surface area, which makes them more soluble, bioavailable, and effective. In terms of bioavailability, it is better to change crystalline drugs into metastable or amorphous forms. During production and storage, however, it is not impossible for the high-energy amorphous or metastable polymorph to change into a low-energy crystal form that is hard to dissolve. It is best to find the drug's polymorph that is the most stable from a thermodynamic point of view. This way, the product can be used in the same way over its shelf life, no matter how it is stored. Particle size reduction: micronization or nanonization is one of the most promising ways to make lipophilic drugs more bioavailable by increasing their surface area and saturation solubility by reducing their size to a submicron level. During the preformulation studies of any formulation, particle size is a very important parameter that should be closely watched. Reducing the size of the particles is a good way to make them more soluble, but if it is not controlled and optimized, it can cause the drug to recrystallize and reaggregate when it is stored. So, a thorough study should be conducted on the size of the particles and how stable they are [2].

By employing nontoxic hydrotropic compounds to solubilize sparingly water-soluble medicinal molecules, there are various options to solve this issue within one method [3]. Carl A. Neuberg, a scientist, invented the term hydrotropy in 1916. Initially, Neuberg claimed that adding certain organic salts might greatly enhance the solubility of hydrophobic compounds. The hydrotrope is the solubilizing agent of a hydrophobic molecule, and the behavior is known as hydrotropy [4]. Hydrotropic molecules are added in certain quantities to increase the solubility of insoluble medicines, referred to as the minimum hydrotropic concentration (MHC). Hydrotropes have both hydrophobic and hydrophilic portions, and they have a low hydrophobic fraction in comparison to the surfactant. The mix of hydrophobic and hydrophilic components determines the success of hydrotrope solubilization. The hydrotropic potency of a molecule increases as the hydrophobic component increases and the load on the hydrophilic part decreases [5]. Hydrotropes use an amphiphilic chemical structure to increase the solubility of sparingly soluble organic compounds in water. By introducing a second solute, this molecular phenomenon improves the aqueous solubility of low-soluble solutes. Hydrotropy is thought to be better than other methods of solubilization, such as miscibility, micellar solubilization, cosolvency, and salting in, because the nature of the solvent is not affected by pH, it has a high level of selectivity, and it does not need to be emulsified. All you have to do is mix the drug with hydrotrope in water. It does not require that hydrophobic drugs be changed chemically, that organic solvents be used, or that an emulsion system be made [6].

Combining two hydrotropic compounds has a significant synergistic impact on improving the solubility of medicines with low water solubility. Mixed hydrotropy will also be employed in the development of aqueous formulations for medicines with low aqueous solubility [5]. Hydrotropic agents can be anionic, cationic, neutral, organic, inorganic, or liquids or solids in the natural world. The hydrotropes having anionic hydrophilic groups have received more attention than the hydrotropes derived from cationic headgroups [7]. The mechanics of hydrotrope have been the subject of several investigative studies. Three designs can be used to condense the available suggested mechanisms:

- The hypothesis of self-aggregation
- The hypothesis of water structure
- Solute hydrotrope hypothesis

Hydrotrope assemblies are distinguished from other solubilizers by their distinct geometrical properties and various association patterns [5]. Many drugs that do not dissolve well in water are easier to dissolve in hydrotropic solutions of sodium benzoate, sodium aciculate, urea, nicotinamide, sodium citrate, and sodium acetate. They do not have a critical concentration where self-aggregation happens all at once. Instead, self-aggregation happens step by step when a solubilizer is added. Hydrotropes are used in the industrial world to make surfactants that are more concentrated. For example, they are used to make detergents [8].

Utilizing solid dispersions has shown to be one of the most effective ways to enhance the release of medications that are not easily soluble. One of the most important strategies for dealing with oral absorption of poorly soluble compounds that is dissolution rate-limited is solid dispersion. According to the physical state of the solid dispersion, the formulation of poorly soluble compounds as solid dispersions may lead to particle size reduction, improved wetting, reduced agglomeration, changeability in the physical state of drug molecules, and perhaps molecular dispersion [9]. A collection of solid products with at least two unique components is referred to as solid dispersion. The hydrophobic medicine and the hydrophilic carrier are generally the two components of solid dispersion. Solid dispersions are often created by the processes of solvent evaporation, solvent fusing, and solvent oxidation [10]. Hydrotropic solid dispersions are commercially viable because they are easy to make on a large scale and the technology is simple. They improve the therapeutic potential of solid oral medications that do not dissolve well in water [5].

**Figure 1:**
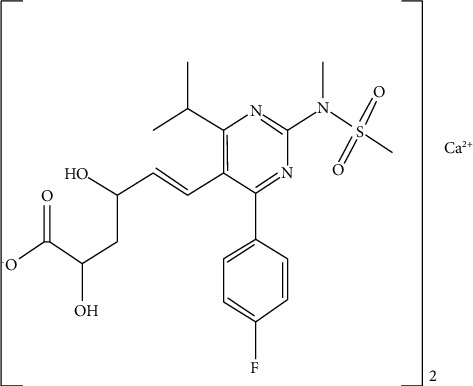
Rosuvastatin calcium molecular structure [11].

Rosuvastatin calcium (ROS) is a cholesterol-lowering drug created by AstraZeneca and licensed in the US in 2003. Statins block 3-hydroxy-3-methylglutaryl-coenzyme A (HMG-CoA) reductase, an enzyme that transforms HMG-CoA into mevalonic acid. In the manufacture of cholesterol, HMG-CoA reductase is a rate-limiting enzyme [11]. Because of its low solubility in water, rosuvastatin has poor solubility in gastrointestinal fluids, and its oral bioavailability is restricted to roughly 20% due to substantial first-pass metabolism [12]. It is *t*1/2, which will be 19 hours [13]. The chemical structure of rosuvastatin calcium is shown in Figure 1.

Due to its limited water solubility, rosuvastatin is categorized as class II in the biopharmaceutics classification system (BCS). Reduced solubility in biological fluids, which results in low bioavailability following oral administration, is one of the medication's most serious issues.

The aims of this study are to improve the solubility and dissolution rate of rosuvastatin calcium, a weakly water-soluble medication, by applying hydrotropy and combined hydrotropic agents in solid dispersion methods. To describe the physiochemical characteristics of the model medication, rosuvastatin calcium, a rotary evaporator, and lyophilization techniques were used to create a solid dispersion of rosuvastatin calcium with the help of sodium benzoate, sodium salicylate, and urea as hydrotropic agents, to confirm the compatibility of pharmaceutical excipients with the solid dispersion. Finally, a comparative analysis of the solubility and dissolution profile of rosuvastatin calcium solid dispersion by rotary evaporator, lyophilization, and physical mixing procedures will be carried out.

## 2. Materials and Methods

### 2.1. Materials

The project involves a lot of different materials. Their origins varied, whether in terms of countries or companies based in the same country. Their chemical types and classifications also differed. Materials used in this investigation are extra pure and are given in Table 1.

### 2.2. Instruments

The instruments being used in this study are available, for the most part, at the College of Pharmacy at Duhok University. Scanning electron microscopy (SEM) is supported by the ULTUM lab at Gaziantep University. Deferential scanning calorimetry (DSC), Fourier transform infrared (FTIR) spectroscopy, and X-ray Diffraction (XRD) were used throughout the study supply by the Çukurova University in Turkey. The instruments used in this study are listed in Table 2.

## 3. Methodology

### 3.1. Determination of Rosuvastatin Calcium Melting Point

The capillary fusion method was used to determine the melting point of rosuvastatin calcium. A small quantity of medication was put into a capillary that was kept inverted and sealed at one end. The electrical melting point device was heated to around 30 degrees below the predicted melting point. The sealed end tube was inserted into the melting point device and heated at a rate of roughly 1-2 degrees per minute until the melting was complete; after that, the melting point was recorded. The melting point determination was conducted in triplicate [13].

### 3.2. Equilibrium Solubility Studies of Rosuvastatin Calcium in Different Hydrotropic Agents

At first, equilibrium solubility investigations were conducted with a variety of hydrotropic agents. Individually, urea (U), citric acid (C), sodium benzoate (SB), sodium salicylate (SS), and mannitol (*M*) aqueous hydrotropic solutions were prepared at concentrations of 10, 20, 30, and 40% w/v. In a 10 ml vial, 5 ml of a specific hydrotropic agent solution was accurately measured, and an excess of rosuvastatin calcium was added and physically agitated for 30 minutes until a saturated solution was generated. To achieve equilibrium solubility, the vial was shaken on a mechanical isothermal bath shaker at 37 ± 0.5°C for 24 hours, and the solution was allowed to equilibrate for another 24 hours. The resulting solution was filtered with a grade of 0.45 *μ*m syringe filter. A portion of the sample was diluted with distilled water and examined at 242 nm using a UV-visibledouble-beam spectrophotometer against the respective solvent as a blank. The equilibrium solubility study was conducted in triplicate [14, 15]. The following formula was used for the calculation of solubility enhancement ratios:

(1) $$\begin{array}{c} \text{Enhancement ratio}=\frac{\text{Solubility of drug in hydrotropic solution}}{\text{Solubility of drug in water}}\text{.} \end{array}$$

Equation (1) is used for the calculation of the solubility enhancement ratio.

### 3.3. Equilibrium Solubility Studies of Rosuvastatin Calcium in Mixed Hydrotropic Solutions

In order to create a clear solution, two to three hydrotropic agents were mixed and dissolved in distilled water. Next, an excessive amount of calcium rosuvastatin was added, and the mixture was shaken until no more rosuvastatin was dissolved. The solutions are mechanically shaken on a mechanical isothermal bath shaker at 37 ± 0.5°C for 24 hours, followed by another 24 hours of equilibration. A grade of 0.45 *μ*m syringe filter was used to filter the resultant solution. Using a UV-visibledouble-beam spectrophotometer, a portion of the material was diluted with distilled water and analyzed at 242 nm. The equilibrium solubility studies were conducted in triplicate. To obtain optimal solubility of rosuvastatin calcium in water, a ratio of three hydrotropic agents was adjusted [14].

### 3.4. Formulation of Hydrotropic Solid Dispersions of Rosuvastatin Calcium by the Solvent Evaporation Technique Using Rotary Evaporator and Freeze-Dryer (Lyophilization) Methods

Each hydrotropic agent, namely, sodium benzoate (SB), sodium salicylate (SS), and urea (U) were carefully weighed (1.333 g) and combined appropriately in a 100 ml beaker for the creation of hydrotropic solid dispersion HSD (1 : 4) ratios. After that, 10 ml of DW was added. On a magnetic stirrer, a solution containing hydrotropic agents was created using Teflon-coated magnetic beads until a clear solution was generated. In the aforesaid solution, a weighed amount of rosuvastatin calcium was dissolved. The solution was then transferred to a rotary evaporator, where the solvents were evaporated for 12 hours at −0.09 MPa pressure and 85°C temperature, using a vacuum pump and a waterbath, which resulted in the formation of solid dispersion by the rotary evaporator (SDRE). The same procedure was followed for the preparation of the solid dispersion by freeze-dryer (SDFD) using the lyophilization method. The resultant solid dispersions were then spread out across many watch glasses and kept in a hot air dry oven at 40 ± 2°C for 24 hours so that any remaining moisture could be quickly evaporated, and a consistent weight could be obtained (due to evaporation). With a glass pestle and mortar, the solid dispersions (SDRE and SDFD) were ground into a fine powder and sieved using a 250 *μ*m (#60) sieve. The resultant solid dispersions were then stored in a desiccated environment for further analysis [14, 16].

### 3.5. Rosuvastatin Calcium Physical Mixtures (PM) Preparation

Drug: hydrotropic agents' ratio of 1 : 4 was used. The components are combined in a glass mortar and pestle for 10 minutes to ensure homogeneity, then passed through a sieve of 250 *μ*m, and kept in a desiccator away from light and humidity until further examination [15].

## 4. Characterization of Solid Dispersions of Rosuvastatin Calcium

### 4.1. Determination of Percent Drug Content

The generated solid dispersions SDRE, SDFD, and physical mixture (PM) containing 10 mg of rosuvastatin calcium and a mixture of hydrotropic agents (sodium benzoate, sodium salicylate, and urea) were carefully weighed and dissolved in 100 ml of 0.05 M citrate buffer pH 6.6 in a 100 ml volumetric flask, yielding a 100 *μ*g/ml solution. The solution was agitated for 30 minutes, filtered using a syringe filter of 0.45 *μ*m, and diluted appropriately with the same solvent before being tested for drug content using a UV-visibledouble-beam spectrophotometer at 241 nm against the respective solvent as a blank [13]. The analysis was carried out three times in each case. The average drug content ± SD was calculated using the following equation:

(2) $$\begin{array}{c} \text{Content }u\text{niformity}=\frac{\text{Actual drug amount in solid dispersion}}{\text{Theor}e\text{tical drug amount in }\text{solid dispersion}}\times 100\text{.} \end{array}$$

Equation (2) is used for determining drug content.

### 4.2. Determination of Percentage Drug Yield

The percentage yield was obtained by weighing the dried solid dispersion and multiplying it by the weight of the beginning components using the following method [17]:

(3) $$\begin{array}{c} \%\;y\text{ield}=\frac{\text{Mass of solid dispersion}}{M\text{ass of drug}+\text{mass of hydrotropic agents}}\times 100\text{.} \end{array}$$

Equation (3) is the formula for calculation of percentage drug yield.

### 4.3. Saturated Solubility Study of Solid Dispersions (SDs) and Physical Mixture (PM)

To assess the solubility of pure rosuvastatin calcium, produced solid dispersions, and physical mixtures in distilled water, saturation solubility tests were performed. The saturation solubility tests were performed in triplicate according to Higuchi and Connors' technique. The excess of solid dispersions and physical mixtures were added to vials containing 10 ml of distilled water. The vials were shaken for 24 hours on a mechanical isothermal bath shaker at 37 ± 0.5°C and then left alone for another 24 hours to equilibrate. A syringe filter with a grade of 0.45 *μ*m was used to filter the drug-containing solution. An aliquot of the filtrate was suitably diluted with the corresponding reagent, and the dilution was analyzed on a UV-visibledouble-beam spectrophotometer [18].

### 4.4. Dissolution Rate Studies of Rosuvastatin Calcium Solid Dispersions

In vitro dissolution of rosuvastatin calcium solid dispersions, physical mixtures, and the pure drug was investigated using a basket stirrer in a USP dissolution apparatus (Industrial Lab) (USP apparatus I method). The dissolution medium was 900 ml of 0.05 M citrate buffer at pH 6.6, which is recommended by the US Food and Drug Administration for dissolution studies with rosuvastatin calcium [12]. The rotation speed was 50 rpm, and the temperature was kept constant at 37 ± 0.5°C during the experiment. Each test employed solid dispersions containing 10 mg of rosuvastatin calcium. At known intervals of time of 5, 10, 15, 20, 25, 30, and 45 minutes, 5 ml of the dissolution medium sample was removed and refilled with the same quantity of medium to maintain a consistent volume throughout the experiment. The withdrawn material was filtered using a 0.45 *μ*m syringe filter. After dilution with a 0.05 M citrate buffer solution, the samples were spectrophotometrically analyzed by detecting the absorbance at 241 nm. The quantity of rosuvastatin calcium released was quantified, displayed against time, and compared to the pure medication. The dissolution studies of each formulation were conducted in triplicate [12, 19].

### 4.5. Fourier Transform Infrared Spectroscopy (FTIR)

A Fourier transform infrared spectrophotometer (FT/IR-6700 type A/JASCO) was used to record FTIR spectra on the sample prepared on KBr disks (2 mg sample in 200 mg KBr). With a resolution of 4/cm, the scanning range was 400–4000/cm. Two forms of hydrotropic solid dispersions of rosuvastatin calcium generated by solvent evaporation using lyophilization and a rotary evaporator, a physical mixture, pure rosuvastatin calcium, sodium benzoate, sodium salicylate, and urea were among the seven samples [20].

### 4.6. Differential Scanning Calorimetry (DSC) Analysis

A differential scanning calorimeter (DSC 3, Mettler Toledo) was used to record thermograms of pure ROS, solid dispersions (SDs), physical mixtures (PM) of the drug, and hydrotropic agents. Each sample was precisely weighed and sealed in an aluminum pan (for the DSC thermograms, 4 mg of the sample was precisely weighed and deposited in an aluminum pan. A glass bell jar was used to cover the pan once it was sealed and put on the heating cell, heating at 10°C each minute.) Under a nitrogen environment (nitrogen flow rate of 20 ml/min), the probes were heated at a rate of 10 K/min from 30 to 300°C [20].

### 4.7. Scanning Electron Microscopy (SEM)

With the help of scanning electron microscopy, we were able to examine the physical structure of the created solid dispersions and describe their shape, as well as verify their particle size (Gemini SEM 300, ZEISS, Europe). A scanning electron microscope equipped with an accelerating voltage ranging from 0.5 to 30 kV was used to picture rosuvastatin, its solid dispersions, and its physical mixture with hydrotropic agents [20].

### 4.8. Powder X-Ray Diffraction PXRD Studies

The powder X-ray diffraction spectra of rosuvastatin were generated using the PANalytical/EMPYREAN X-ray generating system, which was also utilized to construct hydrotropic solid dispersions (HSDs) and physical mixtures (PM). This gives structural information to distinguish and identify polymorphs. The sample was distributed evenly over the graticule, and then, pressure was applied to prevent it from slipping off while maintaining the graticule's vertical position. The graticule was placed in a sample holder and exposed to CuKa-radiation (40 kV, 50 mA). Powder diffraction patterns were created for each sample across the range of 2*θ* from 5 to 40 degrees, using steps of 0.0084 degrees and a counting time of 1.00 degrees min [20].

### 4.9. Statistical Evaluation

All the experiments were repeated three times. Means and standard deviations were used to describe the data (expressed as the mean ± standard deviation). GraphPad InStat Demo software was used to conduct a two-way analysis of variance (ANOVA), which was then used to compare the dissolution release profiles of produced hydrotropic solid dispersions and physical mixing with pure medication. When the value of *P* was less than 0.05, the difference was regarded as statistically significant, whereas when it was more than 0.05, the difference was not considered significant.

## 5. Results and Discussion

### 5.1. Determination of Rosuvastatin Calcium Melting Point

The melting point of rosuvastatin calcium powder used in this study was determined using the melting point apparatus (IA9000, Electrothermal, USA). The melting point of rosuvastatin calcium powder used in this study was in the range of 155–157°C. This range indicates the purity of the product and has a similar melting point range of 156–158 [21] and a melting point of 155°C [22].

### 5.2. Rosuvastatin Calcium Equilibrium Solubility Investigations in Various Hydrotropic Agents and Combined Hydrotropic Agents

Urea, sodium benzoate, and sodium salicylate are the most commonly used hydrotropes that have been reported in the literature to improve the solubility of rosuvastatin calcium [18, 23]. Nevertheless, the solubility values of rosuvastatin calcium in mannitol and citric acid had not been reported. Utilizing pure water as a solvent, the solubility of nevirapine was assessed separately in hydrotropic agent solutions of citric acid and mannitol in the literature reported by Madan et al. [24].

In the current study, the solubility of rosuvastatin calcium was measured in distilled water, obtaining a value of 0.427 ± 0.005 mg/ml.

Various hydrotropic agents such as urea, mannitol, citric acid, sodium benzoate, and sodium salicylate are in different ratios of 10%, 20%, 30%, and 40% w/v. The literature review shows clearly that a drug's ability to dissolve in water increases with hydrotrope concentration. In order to choose appropriate hydrotropes (for sufficient augmentation in solubility) for the weakly water-soluble medication rosuvastatin, relatively high concentrations (10–40%) of hydrotropic agents were tested. The results obtained in this research work could be useful in preformulation studies and dosage form design of rosuvastatin calcium in the pharmaceutical industry.

The drug's solubility enhancement increases with increasing hydrotrope concentration, but the pattern of this rise differs from one hydrotrope to the next, as shown in Table 3. Therefore, in a sodium salicylate solution (40%), the highest solubility was found (125.19 ± 1.86), with an increase in the solubility of rosuvastatin by 292.9 folds. The *P* value is <0.0001, considered extremely significant, and variation among column means is significantly greater than expected by chance.

Complexation with a weak van der Waals attraction such as *π*-*π* or an attractive dipole-dipole interaction is more closely related to how it enhances solubility. The hydroxyl group in sodium salicylate may cause hydrogen bonding and the development of an aggregation product. At lower hydrotrope concentrations, hydrotropic solubilization of rosuvastatin may be owing to weak ionic interactions involving a complexation, but at greater hydrotrope concentrations of 40% w/v, it may be due to molecular aggregation and the inclusion of one in these aggregates [18]. In comparison to Nainwal et al., the effect of various hydrotropes such as sodium acetate, sodium benzoate, and sodium salicylate on the solubility of rosuvastatin was investigated. Sodium salicylate (2.0 M) increases the solubility of rosuvastatin by 55 folds. For that reason, depending on statistical values, sodium salicylate was chosen for further study.

### 5.3. Equilibrium Solubility Studies of Rosuvastatin Calcium in Mixed Hydrotropic Agents

The mixed hydrotropic approach has a remarkable synergistic impact on the solubility of medications that are weakly water-soluble [5]. It lowers the concentration of each hydrotropic substance to reduce negative effects [25]. Salicylate is a very interesting ingredient because it can be used in small amounts as a preservative in cosmetics, drugs, and even food. In fact, a link between salicylate usage and Reye's syndrome has been established, along with additional potential adverse effects such as nausea or stomach discomfort [26].

For that reason, to reduce the concentration of sodium salicylate, different ratios of the sodium salicylate and other hydrotropic agents indicated above were tested to determine the enhancement ratio of the solubility of rosuvastatin calcium, such that the total hydrotropic agent concentration was always 40% w/v. The equilibrium solubility data in Table 4 show that the mixture of two hydrotropic blends were capable of increasing the solubility of rosuvastatin calcium by using a mixed hydrotropic concept.

The blend with the highest solubility enhancement ratio (SS : SB : U) was observed. Furthermore, the solution of mixed hydrotropic agents (SS : SB : U) was investigated by altering the ratios of three hydrotropic agents (sodium salicylate: sodium benzoate: urea) such that maximum solubility may be achieved with the least amount of each hydrotropic agent, hence lowering their toxic potential, as shown in Table 5. Moreover, when compared to distilled water, the blend (SS : SB : U) in the ratio of 13.333 for each hydrotropic agent offered the largest solubility of 191.9 ± 38.63 and the enhancement in the solubility of rosuvastatin was 449 times, very significant at a P value of 0.0001, and much larger than predicted by random variation across column averages. The unique interactions between water molecules and rosuvastatin molecules in the presence of dissolved electrolytes may be largely blamed for the increase in rosuvastatin solubility in an aqueous solution of hydrotropic agents owing to mixed hydrotropy. The solubilization boosters include urea, sodium salicylate, and sodium benzoate. Rosuvastatin is “salting-in” due to its increased solubility in (SS : SB : U) solutions, despite its weak water solubility. As a result, the number of water molecules tied up in the orientation zone decreased as the concentration of hydrotropic agents increased, and the solubility of rosuvastatin correspondingly increased. A similar finding was reported by Singh et al. when they worked on Ibuprofen, as a poorly water-soluble drug [27].

For that reason, depending on the statistical analysis, this optimal combination of hydrotropes (sodium salicylate, sodium benzoate, and urea) was chosen for the creation of solid dispersions.

### 5.4. Formulation of Hydrotropic Solid Dispersions of Rosuvastatin Calcium

Hydrotropic solid dispersions are commonly employed to improve the dissolving rate, solubility, and gastrointestinal absorption of medications that are poorly water-soluble [28]. To further boost the drug's solubility, a hydrotropic solid dispersion of rosuvastatin calcium was created using the solvent evaporation technique, in which the rotary evaporator and lyophilization methods have been used. Solvent evaporation can be used to process a variety of pharmaceuticals and polymers that cannot be employed in the melting processes because of their high melting points [9]. Due to the low temperature needed for the evaporation of solvents, the fundamental benefit of the solvent evaporation approach is that the thermal breakdown of medications or carriers may be avoided [29].

An organic solvent is utilized in the solvent evaporation process to dissolve the substances. A strong dispersion is obtained by using correct evaporation techniques. The main drawbacks of this approach include contamination, increased solvent expense, and residual solvent toxicity. The primary advantage of this approach is that while the medication is insoluble in water, the hydrotropic agent is soluble in water. But when there is a significant amount of a hydrotropic agent in water, the product dissolves. After that, water is removed via evaporation to create a solid mass (solid dispersion). The recommended method is different from the current solvent strategy and offers a new way to use the hydrotropic solubilization phenomenon. Without a hydrotropic agent, water is not a good solvent for drugs with low aqueous solubility. Because they are easy to produce on a large scale and help to boost the therapeutic potential of their weakly water-soluble solid oral medicine forms, hydrotropic solid dispersions are commercially successful. When a hydrotropic solubilization strategy is utilized to achieve the greatest plasma concentration, problems associated with poorly water-soluble drugs, such as a delayed beginning of the action, may be managed by the influence of the solid dispersions of the drug. Compared to the solvent evaporation technique, which permits the use of organic solvents, the hydrotropic approach is more affordable, ecologically friendly, and safe. In order to maximize drug release and, by extension, bioavailability, this approach formulates solid dispersions of medications with low water solubility. Compared to existing formulations that employ costly excipients, the newly created formulations are more affordable [5]. Drug: hydrotropic agents' ratio of 1 : 4 was used to prepare hydrotropic solid dispersion by a solvent evaporation method.

The solvent is removed from solutions by lyophilization (freeze dryer) and also by using a rotary evaporator method, in which the medicine is only subjected to a small amount of heat stress during the production of the solid dispersions, which is a significant benefit of freeze drying. The likelihood of phase separation is, however, significantly reduced as soon as the solution is volatilized, which is the most significant benefit [9]. The lyophilization process is generally associated with low heat stress, low phase separation risk, and the creation of porous as well as fluffy and light powder residue [8].

While the benefit of making solid dispersion using a rotating evaporator is that a thin film of heated solvent is formed and distributed across a large surface as a result of the centrifugal force and the frictional force between the rotating flask's wall and the liquid sample. Bumping is prevented by the forces produced by the rotation [29]. Therefore, the hydrotropic solid dispersion of rosuvastatin calcium was prepared in the ratio of 1 : 4 by a solvent evaporation technique using lyophilization and a rotary evaporator for the removal of the solvents.

### 5.5. Determination of Percent Drug Content

The drug content of all formulations (solid dispersion by the freeze-dryer (SDFD) and solid dispersion by the rotary evaporator (SDRE) as well as physical mixture (PM)) were determined to be within pharmacopeial limits, ranging from 102.339% to 104.963% as shown in Table 6. It implies that the medicine is uniformly distributed within all the powder formulations, implying that the procedures utilized in this investigation for the manufacture of solid dispersions are repeatable. A similar finding was observed in the literature, as reported by Inam et al. and Swathi et al. [30, 31] The drug in the formulation of SDFD has the highest percentage of the drug content (104.963%).

The results of the percentage drug yield for all formulations of solid dispersions using a rotary evaporator (SDRE), lyophilization (SDFD), and physical mixture (PM) were found to be 88.5%, 95.13%, and 95%, respectively. Therefore, the maximum percentage of the practical drug yield was found in the SDFD formulation, i.e., 95.13%, when compared with SDRE formulation. These findings indicated that there was no medication loss throughout the preparation and that the drug content in each batch was consistent. Because one of the advantages of creating solid dispersion by lyophilization is that it is easy to carry and transport, the correct loading amount and content homogeneity are essential [29].

The SDRE formulation did not have a high percentage yield. Because rotary evaporations only work with one sample at a time, which is a big problem, it is possible for some sample types to bump, like ethanol and water when used as solvents, which could cause some of the materials that should be kept being lost. Additionally, the outcome of solid dispersion may be stocked up in the rotary flask, which results in incomplete removal of the formulation from the flask [29].

### 5.6. Saturated Solubility Study

The solubility of rosuvastatin calcium in distilled water was determined to be 0.427 ± 0.005 mg/ml. While, the solubility of the solvent evaporation method made with the rotary evaporator, freeze dryer, and the physical mixture was 3.711 ± 0.166, 3.716 ± 0.316, and 3.586 ± 0.142 mg/ml, respectively, as shown in Table 7. Therefore, the solubility of the drug in all formulations increased significantly compared to that of the pure drug (*P* value is <0.0001), which is considered extremely significant, among which the solid dispersion prepared by the lyophilization SDFD method showed maximum solubility (8.703-fold), which is close to the solid dispersion prepared by the rotary evaporator (SDRE) and physical mixture (PM) (8.69 and 8.399 folds), respectively. The finding in this study is similar to the finding in the literature reported by Viswaja and Bhikshapathi [32].

The drug's particle size is initially decreased to submicron size or to molecular size, in the case of a solid solution, which is one of the mechanisms by which the solubility of the medication was raised. Second, the drug is transformed into an amorphous state, followed by a highly soluble, high-energetic state, and lastly, a dissolved carrier that increases the drug particle's wettability [18].

### 5.7. Dissolution Rate Studies of Rosuvastatin Calcium

**Figure 2:**
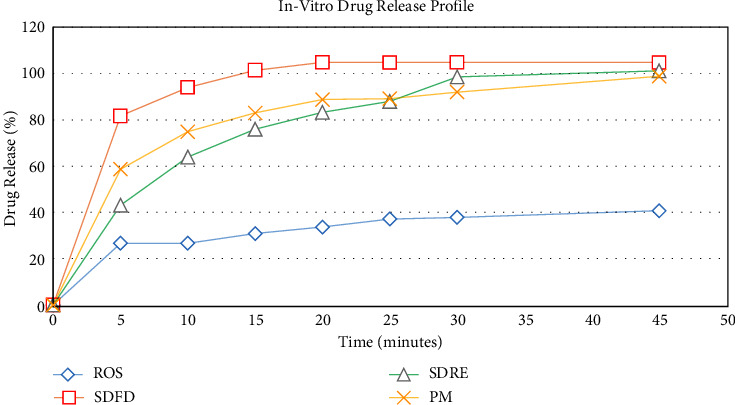
Comparison of the in vitro drug release profiles of pure rosuvastatin calcium ROS and rosuvastatin solid dispersions prepared by a rotary evaporator (SDRE), rosuvastatin solid dispersions prepared by a freeze dryer (SDFD), and rosuvastatin physical mixture (PM).

Various solid dispersions and physical combination formulations were compared to pure drugs for in vitro drug release. Within 45 minutes of dissolution studies, drug release from all produced formulations ranged from 98.83% to 104.78% to pure drug release (40.73 ± 4.44%), as shown in Figure 2. The *P* value is 0.0005, considered extremely significant. A similar finding was demonstrated by the authors of [31].

When the dissolution rates of various formulations were evaluated depending on the technique of preparation, it was revealed that lyophilized hydrotropic solid dispersions had a higher release rate than other formulations with the same concentration of all ingredients. The formulation prepared by lyophilization shows a significant drug release profile (81.68%) after 5 min compared to other formulations, and a similar finding was obtained for a pure drug by Ramu et al. [19].

The formulations prepared by using solid dispersion with lyophilization (SDFD) showed maximum drug release (104.78%) within 45 min. The data were analyzed using an unpaired *t*-test; the two-tailed*P* value is 0.0046, considered extremely significant, and the *t* = 5.730 with 4 degrees of freedom. This demonstrated that the rate of rosuvastatin dissolution increased sharply as the concentration of the hydrotropic agent was raised, and different techniques of preparation were used. Because of the porous and fluffy creation of solid dispersions by lyophilization, the drug released from the formulation SDFD was 3 times more than the pure drug. Due to the drug's reduced crystal size, transformation into the amorphous or microcrystalline state, and increase in wettability, which leads to the formation of a film surrounding the drug particle and thus lowering the drug's hydrophobicity, the dissolution rate of rosuvastatin from solid dispersion by lyophilization has increased significantly [19].

Solid dispersion by rotary evaporator (SDRE), on the other hand, exhibited a 101.18 percent increase in the amount of medicine that is released from the solid dispersion in comparison to the drug in its pure form (40.73%). The two-tailed*P* value of the unpaired *t*-test is 0.0022, which is as regarded very significant. The amount of medication released increased significantly as the concentration of hydrotropic agents increased. This might be attributed to SDRE's high water solubility; it led to better wettability and solubility of drug particles, which ultimately led to improved drug particle dissolution. The rotary evaporator is used to increase bioavailability by boosting solubility and dissolution rates of poorly soluble pharmaceuticals through the production of pharmaceutical complexes and the generation of solid dispersion. This finding agrees with the previous study conducted by Inam et al., 2022, who determined the drug release from the solid dispersion prepared by a rotary evaporator [30].

The physical mixture of rosuvastatin (98.83%) released the drug more significantly than the pure drug (the unpaired *t*-test was conducted and the two-tailedP value <0.0001), which was considered significant, but less than the formulation created using the hydrotropic solid dispersion approaches. These results might be explained by an increase in the surface area of particles, which made greater contact with the dissolving media and resulted in an improved release. The use of hydrotropic agents to solubilize the medication may be responsible for the improvement in dissolution behavior. Because adding more hydrotropic agents to produced formulations increases their solubility and dissolution rate, due to the improved solubility of the prepared formulations, a faster dissolution rate was observed [33].

### 5.8. Fourier Transform Infrared Spectroscopy (FTIR)

**Figure 3:**
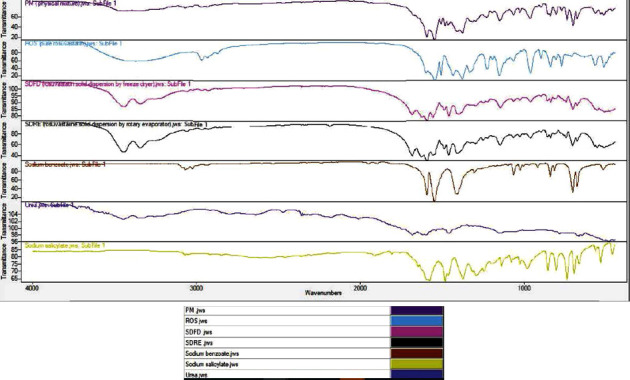
Fourier transform infrared spectroscopy spectrum of pure rosuvastatin ROS, urea, sodium benzoate, sodium salicylate, rosuvastatin physical mixture (PM) (physical combination of rosuvastatin and hydrotropic agents in a ratio of 1 : 4), rosuvastatin solid dispersion by freeze dryer (SDFD) (combination of rosuvastatin and hydrotropic agents in a ratio of 1 : 4), and rosuvastatin solid dispersion by rotary evaporator (SDRE) (combination of rosuvastatin and hydrotropic agents in a ratio of 1 : 4).

Figure 3 of the rosuvastatin calcium FTIR spectrum shows certain distinctive peaks at 3372, 2967, 2933, 1603, 1542, 1509, 1380, 1335, 1229, 1068, 844, and 776 cm^−1^. These peaks are supported by the literature [34]. These peaks represent the intense broad bands at 3372 cm^−1^ and are due to carboxylic O-H stretching, while N-H is suggestive of hydrogen bonding between two molecules of rosuvastatin calcium [30]. Weak absorption bands at 2967 cm^−1^ and 2933 cm^−1^ for C-H stretching sp hybridize the weak stretching band of the carbonyl group (C=O) at 1603 cm^−1^, the aromatic C-N stretching band at 1542 cm^−1^, the C-C stretching band in an aromatic ring at 1509 cm^−1^, the aromatic C–F stretching vibration at 1380 cm^−1^, the sulfone asymmetric stretching band S=O at 1335 cm^−1^, the C-O stretching band in the carbonyl group at 1229 cm^−1^, the S=O stretch at 1068 cm^−1^, the C-F stretch at 844 cm^−1^, and the C-H out of plane bending for aromatic rings at 776 cm^−1^, respectively. The results are in compliance with Butt et al. [12, 30]. The similarities are perceived in the FTIR spectrum of pure rosuvastatin with rosuvastatin physical mixture (PM) and rosuvastatin solid dispersions (SDFD and SDRE). Since peaks linked with rosuvastatin or hydrotropic agents alone have moved into the low range, the spectrum for the physical mixture formulations confirms the occurrence of minor intramolecular interactions between rosuvastatin and hydrotropic agents. The weak interactions are most often indicated by the low magnitude of the peak shift [12]. The reduction in the intensities of the characteristic absorption bands of rosuvastatin was observed in the rosuvastatin solid dispersions formulations (SDFD and SDRE), which might be attributed to the hydrogen bonding interaction between the carboxylic group of rosuvastatin and the hydroxyl group of the hydrotropic agents; this resulted in drug dissolution enhancement, a desired quality given that the complex has to be broken apart to easily release rosuvastatin in vivo. The wavenumber of these shifts may rise or fall. There is an insignificant alteration in the peak pattern of SDFD and SDRE, and no additional peaks were observed, which confirms the lack of chemical interaction within the components of solid dispersions. These findings confirmed that there was a complex formation between drugs and hydrotropic agents but no chemical interaction. The results of the current study were similar to the observations demonstrated by Sarfraz et al., 2017, and Inam et al., [30, 35].

### 5.9. Differential Scanning Calorimetry (DSC)

DSC studies were carried out to see whether there was any interaction between the different components and to examine crystallinity changes. The thermograms of pure compounds were utilized as a baseline for assessing structural changes during the formulation process.

**Figure 4:**
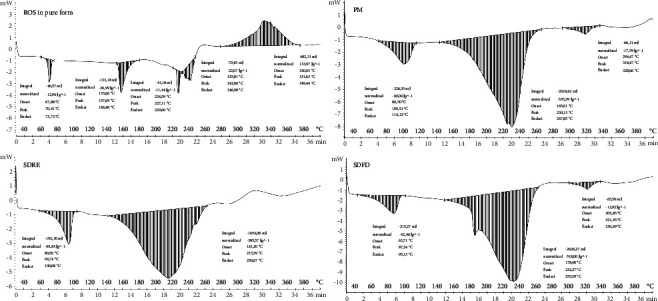
Comparative DSC thermogram of pure rosuvastatin calcium (ROS), rosuvastatin physical mixture (PM) (physical combination of rosuvastatin and hydrotropic agents in a ratio of 1 : 4), rosuvastatin solid dispersions prepared using a freeze dryer (SDFD), and rosuvastatin solid dispersions prepared using a rotary evaporator (SDRE).

The endotherm for onsets of glass transition peaks in the ranges of 68–73°C, with a peak of 70°C perhaps for water loss, was seen in pure rosuvastatin calcium as shown in Figure 4, followed by several endothermic melting peaks in the temperature range of 157.6–242°C. This is due to the polymorphic forms of ROS, which is the main sign of semicrystalline structure and is consistent with other literature findings [30, 36]. The thermograms of the rosuvastatin physical mixture and rosuvastatin solid dispersions (SDFD and SDRE) reveal the shifting of endothermic peaks at 230, 232, and 215°C, respectively, as well as the disappearance of various polymorphic forms of drug peaks. It reveals the endothermic peaks of hydrotropic agents; this means that most of the drug's semicrystalline character is transformed into an amorphous form, resulting in a major shift in the endothermic peaks of the resulting physical mixture and solid dispersions. Any shift or change in melting point or the disappearance of a specific peak indicates that stability has improved.

In the event of solid dispersion by freeze-dryer (SDFD) and rotary evaporator (SDRE), one distinctive endothermic peak was detected at about 87 and 94°C, respectively, due to the loss of water content, and another endothermic peak was seen at 232 and 215°C, correspondingly, that was orienting its melting point. The creation of a solid complex between the drug and the hydrotropic agents was assured by the shifting of peaks. In their research, Mahmood et al. discovered that the removal or shifting of peaks indicated the creation of complexes [35, 37].

### 5.10. Scanning Electron Microscopy (SEM)

**Figure 5:**
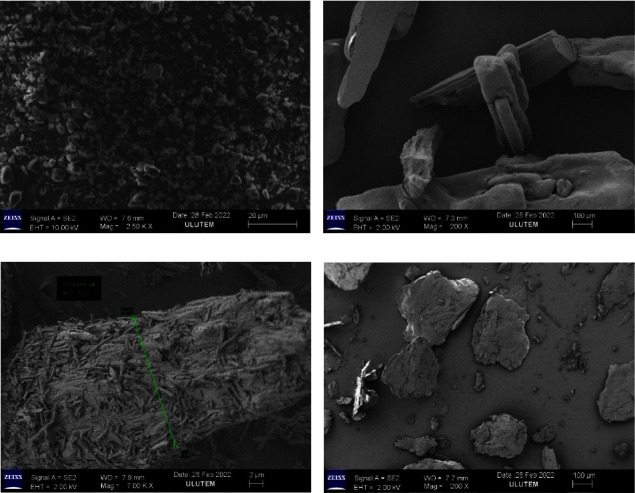
Rosuvastatin calcium scanning electron microscopy image (a), urea (b), sodium benzoate (c), and sodium salicylate (d).

**Figure 6:**
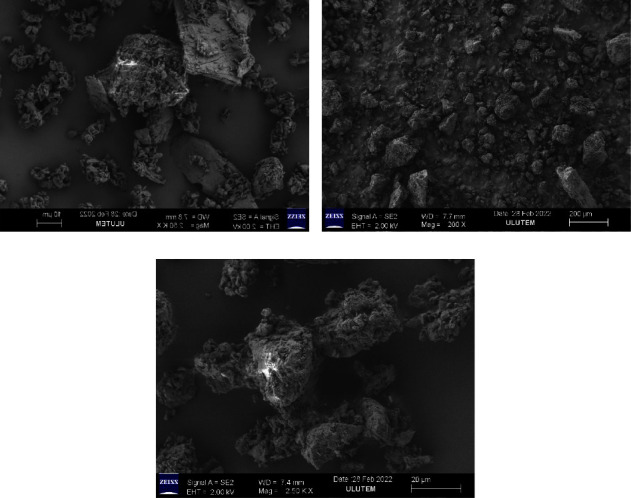
The scanning electron microscopy image of rosuvastatin physical mixture (PM) (physical combination of rosuvastatin and hydrotropic agents in a ratio of 1 : 4) (a), rosuvastatin solid dispersion (rosuvastatin and hydrotropic agents prepared by freeze dryer (SDFD) in a ratio of 1 : 4) (b), and rosuvastatin solid dispersion (rosuvastatin and hydrotropic agents prepared by rotary evaporator (SDRE) in a ratio of 1 : 4) (c).

SEM investigations were carried out on the various components to learn more about their shape and surface morphology. The pure ROS (Figure 5(a)) micrograph shows well-defined tiny crystals with rectangular dimensions and rough edges, which is indicative of their semicrystalline nature [30]. SEM image (Figure 5(b)) had typical rods or elongated crystals of urea, while the SEM image of sodium benzoate (Figure 5(c)) had an aggregate of rode-shape crystals, and the surface morphology (Figure 5(d)) showed that sodium salicylate consisted of cubic-shaped, long, and irregular crystals. The microscopic structure of the produced formulations and physical mixtures differed significantly. The physical mixture of the drug and the hydrotropic agents, as shown in Figure 6(a), showed the existence of the drug in semicrystalline form and displayed differentiation in the manner in which smaller ROS particles seemed to be adhere to larger hydrotropic agents' particles. In solid dispersion formulations, a decrease in crystallinity was found. The freeze dryer and rotary evaporator techniques were used for creating solid dispersions preparation, resulting in smaller formulations. The solid dispersion (SDFD) formulation (Figure 6(b)) prepared by lyophilization revealed that the dispersions were highly porous, loosely networked, friable, and low-density solid form. The finely divided almost amorphous form was noticed in the solid dispersion (SDRE) formulation (Figure 6(c)) generated by a rotary evaporator. The same conclusion was reached by Ramu et al. in his study [19]. Because the surface morphology was porous, there was an increased inflow and intake of media, which resulted in quicker drug release and dissolution, and this was supported by Mahmood et al. in his study [38].

Rosuvastatin was homogeneously diffused into the hydrotropic agents, based on the surface morphology of the solid dispersions in the SDFD and SDRE formulations. According to SEM images, the individual surface properties of hydrotropic agents and rosuvastatin were lost throughout the rotary evaporator and freeze dryer, which might be due to the production of efficient solid dispersion systems. These results showed that the drug was extensively mixed in the carriers, resulting in crystallinity loss. The solid dispersions (SDFD and SDRE) formulations exhibited a significant change in the shapes and particles appeared regular in the shapes with smooth surfaces, a probability due to the complete miscibility of the drug and hydrotropic agents, which supports the result obtained from FTIR and DSC.

### 5.11. Powder X-Ray Diffraction (PXRD) Studies

**Figure 7:**
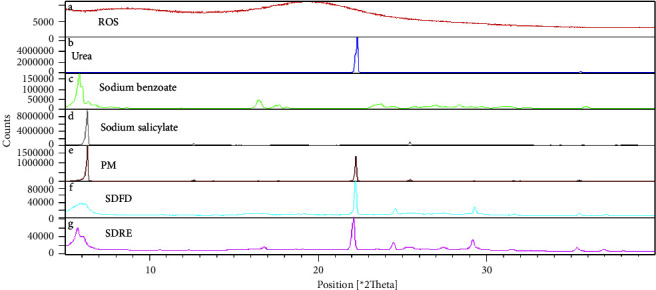
A comparative X-ray diffractogram of pure rosuvastatin ROS, hydrotropic agents (urea, sodium benzoate, and sodium salicylate), rosuvastatin solid dispersions (SDRE and SDFD), and rosuvastatin physical mixture (PM).

Powder X-ray diffractogram (Figure 7) was taken to know about the nature of rosuvastatin, whether it was crystalline or amorphous; urea, sodium benzoate, sodium salicylate, rosuvastatin physical mixture (PM), rosuvastatin solid dispersion prepared using a freeze dryer (SDFD), and rosuvastatin solid dispersion prepared using a rotary evaporator (SDRE). The XRD pattern of pure drug demonstrated that pure ROS has indicated the semicrystalline nature of the drug. Sharp crystalline peaks at 2*θ* diffraction angles are at 8.14, 9.77, and 19.66. Numerous distinct diffraction peaks are present at 2*θ* values 22.2, 29.2, 31.7, and 35.4 in the XRD spectrum of pure urea, whereas sodium benzoate showed a sharp peak at 2*θ* values 5.81, 16.4, 23.7, and 28.33, and sodium salicylate also shows the intrinsic diffraction peaks at 2*θ* values 6.16, 12.5, 25.4, and 31.9 in the XRD spectrum, which indicates that all hydrotropic agents were present as crystalline materials.

The XRD pattern of the physical mixture of rosuvastatin shows a drop in the number of peaks, which is likely due to a decrease in crystallinity. On the other hand, the spectrum of solid dispersions produced by the rotary evaporator (SDRE) and freeze dryer (SDFD) was characterized by a decrease in the number of diffraction peaks. When contrasted with the SDRE and physical mixture, the SDFD XRD pattern has a few wide low-intensity peaks. All the diffraction peaks were caused by the hydrotropic agents' crystals, and no rosuvastatin calcium diffraction peaks were found in the solid dispersions or physical mixture, and similar results were obtained by Ramu et al. in their study [19].

This means that the semicrystalline state of rosuvastatin calcium was modified to an amorphous state, and no new peaks occurred in the XRD spectrums in solid dispersion systems. It is reasonable to assume that the development of hydrotropic solid dispersions or physical mixtures does not lead to any physical or chemical interaction between rosuvastatin calcium and hydrotropic agents since the same peaks at 2*θ* have been shown by the rosuvastatin solid dispersions SDRE, SDFD, and physical mixture, which are characteristic of hydrotropic agents [39].

It is commonly accepted that if three successive relative intensity percentage values in an XRD pattern fall, the samples' crystallinity has decreased, and therefore, these findings can be considered a confirmation of the crystallinity reduction and consequently phase transition. The increase in solubility and bioavailability is attributed to an amorphous system, according to reports [24]. Because amorphous forms are more soluble than crystalline forms, rosuvastatin dissolution was improved in solid dispersions prepared with a rotary evaporator and freeze dryer as compared to a physical mixture and pure drug.

## 6. Conclusion

Due to its limited water solubility, the commonly used antihyperlipidemic HMG-CoA reductase inhibitor, rosuvastatin calcium, has a low and variable oral bioavailability of only 20%. The solubility enhancement of rosuvastatin calcium by using hydrotropic agents and a combination of hydrotropic agents to create a solid dispersion by solvent evaporation technique using a rotary evaporator and lyophilization methods showed a significant enhancement in solubility and dissolution rate in comparison to the pure drug. This study provides evidence for the efficacy of hydrotropy in improving the solubility of medications with low water solubility. The quick dissolution of the sparingly soluble medication rosuvastatin in 0.05 M sodium citrate buffer as a dissolution medium suggests that it has the potential to solubilize the drug in biological fluids, leading to noticeably improved bioavailability as well as a faster onset of action. By dissolving the drug in nonionized form, the concept of mixed hydrotropic solid dispersion is a novel, efficient, and economical method for enhancing the bioavailability of pharmaceuticals that are simply weakly water-soluble. The fact that rosuvastatin's solubility has significantly improved suggests that it may eventually be used to treat other drugs with poor water solubility in cases where low bioavailability is a serious issue.

## Figures and Tables

**Table 1:** Materials and their suppliers that are used in the study.

SI no.	Ingredients	Suppliers
1	Rosuvastatin calcium	Awamedica Drug Industries, Iraq
2	Sodium benzoate	AZ Chem, Jordan
3	Sodium salicylate	AZ Chem, Jordan
4	Urea	AZ Chem, Jordan
5	Mannitol	AZ Chem, Jordan
6	Citric acid	AZ Chem, Jordan
7	Dibasic sodium phosphate	AZ Chem, Jordan
8	Methanol	Scharlau, Spain

**Table 2:** The instruments with their models/companies.

No.	Instruments	Model/company
1	UV-visible spectrophotometer	6850, Jenway.
2	Tablet dissolution tester	HM L-TT-DS61, Phaemachine, China.
3	Magnetic hot plate and stirrer	US151, Stuart, UK.
4	Digital pH meter	pH700, EUTECH, Singapore.
5	Electronic analytical balances	AZ, Sartorius, Germany.
6	Scanning electron microscope (SEM)	GeminiSEM 300, ZEISS, Europe.
7	Hot air oven	100–800, Memmert, Germany.
8	Rotary evaporator machine	RE300, Stuart, UK.
9	Freeze dryer machine	HM L-L-Y-TP8 freeze dryer, China.
10	Digital melting point apparatus	IA9000, Electrothermal, USA.
11	Fourier transform infrared (FTIR)	FT/IR-6800, JASCO, Japan.
12	Deferential scanning calorimetry (DSC)	DSC3, Mettler Toledo.
13	Shaking water bath	SBS40, Stuart, UK.
14	X-ray diffraction (XRD)	PANalytical/EMPYREAN.
15	Water distillation assembly	D Lab Tech, Korea.
16	Rotary evaporator bath	RE300OB, Stuart, UK.
17	Vacuum pump	RE3022C, Stuart, UK.

**Table 3:** Rosuvastatin calcium equilibrium solubility in various hydrotropic agents such as urea, mannitol, citric acid, sodium benzoate, and sodium salicylate in different ratios of 10%, 20%, 30%, and 40% w/v.

Hydrotropic agents	Concentration 10% W/V	Concentration 20% W/V	Concentration 30% W/V	Concentration 40% WV	Solubility enhancement ratio
Urea	0.85 ± 0.07*∗*	1.06 ± 0.08	1.13 ± 0.16	1.36 ± 0.10	3.198
Mannitol	0.49 ± 0.13	0.599 ± 0.09	0.66 ± 0.10	0.89 ± .028	2.085
Citrate acid	0.45 ± 0.003	0.75 ± 0.05	0.78 ± 0.1	0.93 ± 0.17	2.192
Sodium benzoate	22.91 ± 1.46	39.03 ± 4.53	102.66 ± 14.1	118.6 ± 13.8	277.556
Sodium salicylate	26.97 ± 1.04	49.92 ± 2.58	97.32 ± 4.13	125.19 ± 1.8	292.961

*∗*Solubility in mg/ml; U, urea; M, mannitol; C, citric acid; SB, sodium benzoate; SS, sodium salicylate. The information is presented as the mean ± SD (*N* = 3).

**Table 4:** The outcomes of the rosuvastatin calcium equilibrium solubility in a mixture of two hydrotropic agents with a total concentration of 40%.

Combination	Total concentration (% W/V)	Individual concentration (% W/V)	Solubility, mg/ml	Solubility enhancement ratio
SS*∗* : U*∗*	40	20 : 20	77.62 ± 2.31	181.65
SS : C*∗*	40	20 : 20	32.48 ± 0.52	76.015
SS : M*∗*	40	20 : 20	54.98 ± 2.72	128.66
SS : SB*∗*	40	20 : 20	179.92 ± 40.05	421.02
SS : SB	40	5 : 35	81.10 ± 1.54	189.78
SS : SB	40	10 : 30	51.29 ± 3.38	120.01
SS : SB	40	15 : 25	166.40 ± 7.24	389.37

*∗*SS, sodium salicylate; U, urea; C, citric acid; M, mannitol; SB, sodium benzoate. The information is presented as the mean ± SD (*n* = 3).

**Table 5:** The results of equilibrium solubility of rosuvastatin calcium in a mixture of three hydrotropic agents of different ratios with a total concentration of 40%.

Combination	Total concentration (%W/V)	Individual concentration (%W/V)	Solubility, mg/ml	Solubility enhancement ratio
SS : SB : M	40	13.333*∗*	73.81 ± 1.49	172.722
SS : SB : C	40	13.333	47.86 ± 1.43	112.005
SS : SB : U	40	13.333	191.94 ± 38.63	449.137
SS : SB : U	40	20 : 10 : 10	100.6 ± 12.7	235.414
SS : SB : U	40	20 : 15 : 05	128.77 ± 4.02	301.328
SS : SB : U	40	20 : 05 : 15	89.87 ± 5.18	210.314
SS : SB : U	40	10 : 15 : 15	85.58 ± 1.74	200.265

*∗*Each hydrotropic agent 13.333. The data are presented as the mean ± SD (*n* = 3).

**Table 6:** Determination of the percentage drug content of rosuvastatin solid dispersions SDRE, SDFD, and physical mixture.

NO SI	Solid dispersion	% drug content
1	SDRE*∗*	102.339 ± 1.378
2	SDFD*∗*	104.963 ± 0.149
3	PM*∗*	103.154 ± 0.605

*∗*SDRE, rosuvastatin solid dispersion by the rotary evaporator; SDFD, rosuvastatin solid dispersion by the freeze dryer; PM, rosuvastatin physical mixture. The data are presented as the mean ± SD (*n* = 3).

**Table 7:** Saturated solubility data of rosuvastatin calcium in pure form and prepared formulations (rosuvastatin solid dispersion using freeze dryer (SDFD), rosuvastatin solid dispersion by rotary evaporator (SDRE), and rosuvastatin physical mixture (PM)).

SI no.	Formulation	Solubility, mg/ml	SER*∗*
1	ROS*∗*	0.427 ± 0.005	--------
2	SDRE	3.711 ± 0.166	8.690
3	SDFD	3.716 ± 0.316	8.703
4	PM	3.491 ± 0.03	8.177

*∗*ROS, pure rosuvastatin calcium; SER, solubility enhancement ratio.

## Data Availability

The data used to support this study are included within the article.
